# Supplementary material for: Genetic Distinctiveness Highlights the Conservation Value of a Sicilian Manna Ash Germplasm Collection Assigned to Fraxinus angustifolia (Oleaceae)
Source: Plants (Basel). 2020 Aug 14;9(8):1035. doi: 10.3390/plants9081035 (PMC7463994; doi:10.3390/plants9081035)

**Figure S1** Neighbor-Joining (NJ) tree based on population pairwise fixation index. Genetic distances were computed among populations grouped based on Country of origin.


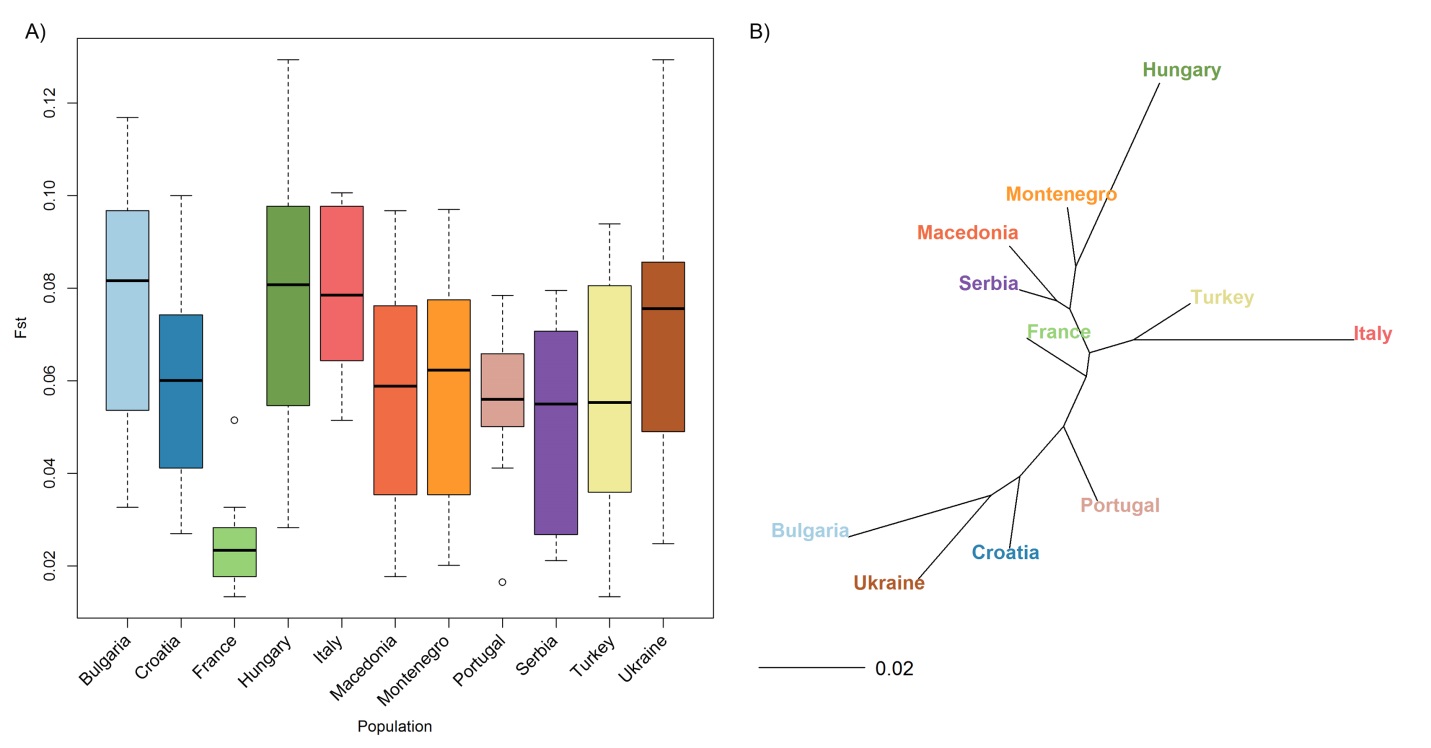

Supplement: Supplementary file 1 [file plants-09-01035-s001.zip › Supplementary_files/Figure S1.docx]
