# Supplementary material for: Genetic Distinctiveness Highlights the Conservation Value of a Sicilian Manna Ash Germplasm Collection Assigned to Fraxinus angustifolia (Oleaceae)
Source: Plants (Basel). 2020 Aug 14;9(8):1035. doi: 10.3390/plants9081035 (PMC7463994; doi:10.3390/plants9081035)
Supplement: Supplementary file 1 [file plants-09-01035-s001.zip › Supplementary_files/Table S1.docx]

**Table S1** Morphological traits of studied samples, belonging to manna ash collection and samples belonging to *F. ornus*, used as reference. In bracket the number of samples analysed belonging to each local variety/species. Three replicates for each trait were analysed.

| **Local variety/species** | **WLL range (cm)^*^** | **N° of leaflets^*^** | **Petiole length (mm)^*^** | **Rachis length (mm)^*^** | **Leaf shap** | **Leaf margin** | **Fruit length range**  **(cm)^*^** | **Fruit width**  **(cm)^*^** | **Seed length range**  **(cm)^*^** | **Seed width/length ratio^*^** |
| --- | --- | --- | --- | --- | --- | --- | --- | --- | --- | --- |
| Abbassa cappeddu (3) | 12.33 | 5 | 1.33 | 5.66 | cuneate | continuous | 5 | 0.8 | 0.9 | 0.28 |
| Baciciu (4) | 16.25 | 5.75 | 1.50 | 12.25 | cuneate | dentate | 5.25 | 0.80 | 1.65 | 0.22 |
| Cavolo (3) | 15 | 8 | 3 | 10 | elliptic | continuous | 4.97 | 0.8 | 2.37 | 0.21 |
| Macigna (3) | 19.5 | 8.7 | 3.3 | 8 | attenuate | continuous | 3.9 | 0.83 | 1.8 | 0.21 |
| Nivuru (3) | 13.8 | 7 | 4.5 | 8.3 | attenuate | dentate | 4.1 | 0.87 | 2 | 0.22 |
| Nsiriddu (3) | 14.1 | 6.3 | 3.7 | 7 | elliptic | dentate | 4.4 | 0.8 | 1.5 | 0.24 |
| Russu (3) | 18 | 9 | 2.33 | 7 | attenuate | cont/dent | 4.2 | 0.8 | 1.7 | 0.24 |
| Sarvaggio (3) | 12.5 | 6 | 4.66 | 8 | attenuate | dentate | 4.33 | 0.83 | 1.9 | 0.2 |
| Verdello (7) | 12.78 | 5.28 | 1.57 | 5.85 | cuneate | continuous | 3.5 | 0.8 | 1.4 | 0.27 |
| Frassino monumentale (1) | 22.25 | 6 | 4 | 12 | attenuate | continuous | 4.4 | 0.85 | 0.85 | 0.195 |
| Nsiriddu monumentale (1) | 16.1 | 6 | 3.5 | 11 | elliptic | dentate | 4.4 | 0.8 | 1.5 | 0.24 |
| *mean* | *15.69* | *6.64* | *3.04* | *8.64* | *-* | *-* | *4.40* | *0.82* | *1.60* | *0.23* |
| *F. ornus* (4) | 21 | 5.5 | 4.25 | 13.5 | attenuate | continuous | 4.15 | 0.85 | 0.85 | 0.18 |

^*^ Mean of values recorded; WLL: Whole Leaf Length.
