# Supplementary material for: Genetic Distinctiveness Highlights the Conservation Value of a Sicilian Manna Ash Germplasm Collection Assigned to Fraxinus angustifolia (Oleaceae)
Source: Plants (Basel). 2020 Aug 14;9(8):1035. doi: 10.3390/plants9081035 (PMC7463994; doi:10.3390/plants9081035)
Supplement: Supplementary file 1 [file plants-09-01035-s001.zip › Supplementary_files/Table S3.docx]

**Table S3** Allelic Richness (*AR*) evaluated for FAN accessions grouped based on sampling location. In the table geographic coordinates (Long and Lat) and *AR* values for each nSSR were indicated.

| **Sample** | **Long** | **Lat** | **Fem4** | **Fem11** | **Fem12** | **Fem16** | **Fem19** | **M230** | **SSR326** | **SSR427** | **SSR431** | **SSR520** | **SSR528** |
| --- | --- | --- | --- | --- | --- | --- | --- | --- | --- | --- | --- | --- | --- |
| Bulgaria | 28.06201 | 43.35801 | 2.435 | 4.335 | 2.838 | 1.982 | 2.513 | 4.394 | 1.345 | 2.655 | 2.655 | 1.746 | 1.976 |
| Croatia1 | 15.96901 | 43.81901 | 3.589 | 4.490 | 3.493 | 1.345 | 1.690 | 4.211 | 2.674 | 3.301 | 1.746 | 2.274 | 3.171 |
| Croatia2 | 16.70601 | 45.41801 | 2.513 | 3.188 | 3.068 | 1.746 | 1.690 | 3.972 | 2.261 | 3.027 | 1.746 | 2.857 | 2.533 |
| France1 | -0.84001 | 45.36901 | 2.961 | 2.032 | 4.181 | 2.285 | 4.046 | 4.540 | 2.537 | 3.455 | 1.643 | 3.092 | 3.816 |
| France2 | 3.48501 | 43.28001 | 3.816 | 3.293 | 4.675 | 2.285 | 4.445 | 4.175 | 2.285 | 2.773 | 1.950 | 2.961 | 3.067 |
| France3 | 3.11501 | 43.39401 | 3.266 | 4.518 | 3.906 | 2.724 | 2.604 | 4.356 | 2.330 | 3.271 | 1.956 | 2.933 | 3.011 |
| France4 | 2.99401 | 43.24101 | 3.199 | 3.450 | 4.106 | 2.315 | 3.356 | 4.316 | 1.851 | 2.435 | 2.168 | 1.956 | 2.749 |
| France5 | 6.53701 | 43.23701 | 3.433 | 3.205 | 3.723 | 1.584 | 2.196 | 3.723 | 2.330 | 2.435 | 1.584 | 2.749 | 4.001 |
| France6 | 3.03301 | 42.58601 | 3.244 | 4.384 | 3.933 | 3.250 | 3.222 | 2.724 | 1.929 | 2.330 | 2.330 | 2.933 | 3.561 |
| France7 | 1.22601 | 43.64101 | 2.499 | 3.539 | 3.484 | 1.345 | 2.034 | 4.067 | 2.533 | 1.917 | 2.091 | 1.851 | 2.499 |
| Hungary | 19.23101 | 47.26801 | 1.976 | 2.985 | 2.961 | 1.984 | 2.337 | 2.721 | 1.984 | 2.600 | 1.000 | 1.976 | 1.984 |
| Italy1 | 15.67201 | 38.22201 | 3.644 | 3.902 | 3.307 | 3.437 | 2.539 | 4.085 | 1.180 | 1.332 | 1.000 | 4.181 | 3.231 |
| Italy2 | 14.01602 | 37.88301 | 3.388 | 3.693 | 3.645 | 1.345 | 3.589 | 4.067 | 1.345 | 1.851 | 1.956 | 2.168 | 3.071 |
| Macedonia | 22.49101 | 41.17201 | 3.583 | 3.962 | 4.394 | 1.746 | 3.250 | 4.211 | 2.749 | 3.327 | 1.345 | 3.618 | 3.400 |
| Montenegro | 19.34801 | 41.86601 | 2.857 | 3.222 | 3.894 | 2.196 | 2.274 | 4.912 | 2.435 | 2.749 | 1.746 | 3.618 | 2.943 |
| Portugal1 | -7.66301 | 39.19301 | 3.189 | 3.559 | 3.852 | 2.424 | 3.227 | 4.514 | 2.385 | 2.646 | 1.940 | 1.862 | 3.255 |
| Portugal2 | -7.67501 | 41.25501 | 3.972 | 2.330 | 3.693 | 2.594 | 3.188 | 4.490 | 1.584 | 2.911 | 1.000 | 2.793 | 1.584 |
| Serbia1 | 20.18301 | 44.67801 | 3.872 | 4.702 | 4.624 | 1.584 | 2.943 | 4.546 | 2.674 | 3.244 | 1.345 | 2.491 | 3.506 |
| Serbia2 | 22.59701 | 44.15401 | 3.488 | 4.514 | 4.326 | 2.158 | 1.446 | 4.398 | 2.717 | 3.076 | 1.000 | 2.868 | 3.443 |
| Turkey | 38.58801 | 37.91102 | 3.317 | 4.636 | 4.061 | 2.545 | 3.317 | 3.317 | 1.998 | 2.741 | 1.773 | 2.545 | 2.741 |
| Ukraine | 33.60201 | 44.50601 | 3.867 | 4.490 | 2.499 | 1.851 | 1.929 | 3.171 | 1.345 | 3.205 | 2.499 | 2.540 | 3.011 |
